# An optimal stratified Simon two‐stage design

**DOI:** 10.1002/pst.1742

**Published:** 2016-03-02

**Authors:** Deepak Parashar, Jack Bowden, Colin Starr, Lorenz Wernisch, Adrian Mander

**Affiliations:** ^1^ Statistics and Epidemiology Unit, and Cancer Research Centre, Division of Health Sciences University of Warwick Coventry UK; ^2^ MRC Biostatistics Unit Hub for Trials Methodology Research Coventry UK

**Keywords:** Stratified Design, Adaptive Enrichment, Phase II Oncology

## Abstract

In Phase II oncology trials, therapies are increasingly being evaluated for their effectiveness in specific populations of interest. Such targeted trials require designs that allow for stratification based on the participants' molecular characterisation. A targeted design proposed by Jones and Holmgren (JH) Jones CL, Holmgren E: ‘An adaptive Simon two‐stage design for phase 2 studies of targeted therapies’, Contemporary Clinical Trials 28 (2007) 654‐661.determines whether a drug only has activity in a disease sub‐population or in the wider disease population. Their adaptive design uses results from a single interim analysis to decide whether to enrich the study population with a subgroup or not; it is based on two parallel Simon two‐stage designs. We study the JH design in detail and extend it by providing a few alternative ways to control the familywise error rate, in the weak sense as well as the strong sense. We also introduce a novel optimal design by minimising the expected sample size. Our extended design contributes to the much needed framework for conducting Phase II trials in stratified medicine. © 2016 The Authors Pharmaceutical Statistics Published by John Wiley & Sons Ltd

## 1 Introduction

Group‐sequential trial designs, in which the data are periodically assessed to determine whether the trial should continue, can be far more efficient than trials of a fixed sample size. They help in minimising the trials' duration, cost and number of people exposed to ineffective treatments 1, 2. The simplest example of an adaptive trial is a two‐stage design introduced for Phase II cancer trials by Gehan 3, Fleming 4, Simon 5 and many others 6, 7. Of particular interest is the Simon two‐stage design 5; it tests a single treatment with a binary response, and an interim analysis is used to allow the trial to stop early for futility only. Simon's design requires pre‐specification of the null response rate, the desired type I error probability and sufficient power at a targeted response rate. Assuming the null hypothesis to be true, it minimises the expected sample size and is, therefore, *optimal*. If it minimises the total sample size, then Simon's design is referred to as a *minimax* design.

In recent years, there has been a concerted effort to tailor treatment (especially cancer therapy) to the specific needs of patients, so that they are most effective. This is the guiding principle underlying *stratified medicine* 8, 9. A patient's biomarker(s) (a general term for a genetic or bio‐chemical measurement) are increasingly being used to define the treatment subgroup to which they should belong. This presents a challenge for clinical trials: conventional (and even adaptive) trial designs aim to estimate a common treatment effect in the disease population. In the realm of stratified medicine, designs are needed to both assess the clinical utility of biomarkers as a diagnostic tool to guide treatment, as well as to estimate a treatment's effect within each biomarker subgroup.

Various designs have been proposed for the biomarker trials, for example biomarker‐stratified designs, enrichment designs and the biomarker‐strategy designs 10, 11, 12, 13, 14, 15, 16, 17, 18. The reader is referred to 19 for a comprehensive review. The execution of biomarker trials often requires interim monitoring and analysis. Therefore, it is natural to set them in the context of an adaptive design. In this paper, we review and extend a biomarker stratified Simon two‐stage design proposed by Jones and Holmgren (JH) 20 in the context of Phase II cancer trials, which is now briefly described. In the first stage of the JH design, a new therapy is assessed for its activity (its response rate) simultaneously in the biomarker‐positive and biomarker‐negative sub‐populations. The JH design then uses the first stage data to guide whether to (a) continue to study an unselected (biomarker positive and negative) population during the second stage, or (b) enrich the population by enrolling only biomarker‐positive subjects. This design has been used in a Phase II study of HER2‐negative breast cancer 21. In Section 2, we discuss the JH design framework in detail. In Section 3, we provide explicit formulae for probabilities of various positive outcomes and extend the JH framework so that error rates can be controlled using several new definitions. In Section 4, we report optimal designs for the various error rate definitions, and we conclude with a discussion in Section 5.

## 2 Summarising Jones–Holmgren Design

The purpose of the Jones–Holmgren (JH) design 20 is to assess the performance of an experimental treatment in a biomarker‐negative population, and potentially a biomarker‐positive population as well. Let the true response rates for the *biomarker‐negative* and *biomarker‐positive* sub‐populations be *p* ^−^ and *p* ^+^, respectively. The null hypotheses are $H_{0}^{-}\;:\;p^{-}\;=\;p_{0}^{-},H_{0}^{+}\;:p^{+}\;=\;p_{0}^{+}$, and the alternative hypotheses are $H_{1}^{-}:p^{-}=p_{1}^{-},H_{1}^{+}:p^{+}=p_{1}^{+}$ where $p_{1}^{-}>p_{0}^{-}$ and $p_{1}^{+}>p_{0}^{+}$. This hypothesis setup implies that any response rate *p*_1_>*p*_0_ (*i.e.* a positive outcome) is considered effective and warrants further study, that is, a *go* decision, whereas any response rate *p*_1_≤*p*_0_ is considered ineffective and constitutes a *no‐go* decision. While this would be true at the second stage, stopping the study at the first stage as a no‐go tends not to be a conclusion of *p*_1_≤*p*_0_ but rather ruling out that the response rate is as good as *p*_1_. We further fix $p_{0}^{-}\;=\;p_{0}^{+}$. This implies that the biomarker is potentially *predictive* of treatment effect, rather than a *prognostic* indicator of underlying health. For the particular example trial, they consider $p_{0}^{+}\;\;=\;p_{0}^{-}=0.03,p_{1}^{+}=0.15,p_{1}^{-}=0.10$. An order restriction is assumed for the response rates, namely, *p* ^−^≤*p*_*u*_≤*p* ^+^ (i.e. *p*_*u*_ is the response rate in the unselected population which is a weighted average of the response rates in the biomarker‐negative and biomarker‐positive sub‐populations), and we stick to this assumption in this paper. The clinical reason behind such an order restriction is that the biomarker‐positive subjects are expected to be more sensitive to an interventional targeted drug being developed than the biomarker‐negative subjects; this has been the assumption not only in the example trial considered by JH but also in a recent biomarker‐stratified phase II trial REMAGUS 02 22 for large operable and locally advanced breast cancer setup according to two parallel two‐stage Fleming design 12.

**Figure 1:**
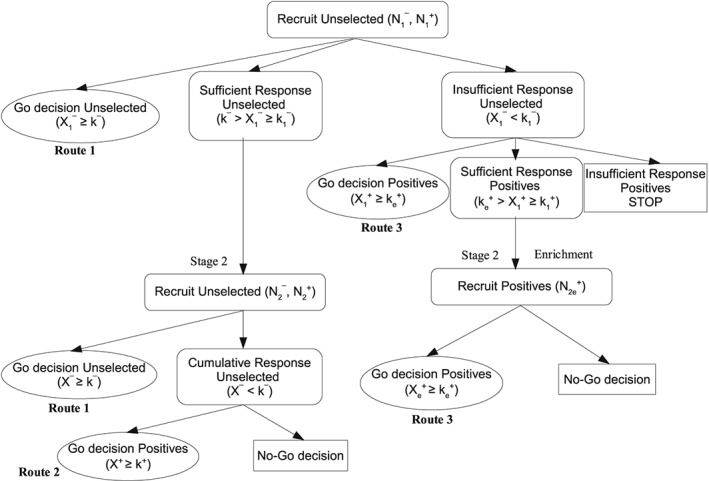
A schematic for the adaptive enrichment stratified design.

At Stage 1, they begin with two parallel studies (Figure 1), one in $N_{1}^{-}$ biomarker‐negative participants and one in $N_{1}^{+}$ biomarker‐positive participants (the total sample size of the first stage is $N_{1}=N_{1}^{-}+N_{1}^{+}$). Activity is first assessed in the biomarker‐negative sub‐population and, if present, continues to Stage 2 recruiting a further *N*_2_ participants in the unselected population. However, if no activity is indicated in the biomarker‐negative sub‐population at Stage 1, they then assess activity in the biomarker‐positive sub‐population and in case of an indication of activity continue to Stage 2 recruiting a further $N_{2e}^{+}$ participants (subscript *e* denotes *enrichment*) in the same sub‐population and subsequently test for a positive outcome or a go decision.

On the other hand, in the earlier case of recruiting further participants in the unselected population at Stage 2, that is, $N_{2}=N_{2}^{-}+N_{2}^{+}$ (where $N_{2}^{-}$ and $N_{2}^{+}$ are the number of Stage 2 biomarker‐negative and biomarker‐positive participants, respectively, and JH assuming the prevalence of marker‐positive subjects to be 40*%*), they test for a positive outcome in the biomarker‐negative sub‐population, and in case of sufficient responders, the treatment is declared effective in the biomarker‐negative sub‐population. Note that due to the order restriction, the treatment can also be immediately declared effective in the biomarker‐positive population, without the need for further testing. If, however, the treatment is ineffective in the biomarker‐negative population at Stage 2, they then test for a positive outcome in the biomarker‐positive sub‐population. Furthermore, let $N^{-}\;\;=\;N_{1}^{-}\;+\;N_{2}^{-},N^{+}\;\;=\;N_{1}^{+}\;\;+\;N_{2}^{+}$, and $N_{e}^{+}\;\;=N_{1}^{+}\;+\;N_{2e}^{+}$. We amend the JH design in that we allow the trial to stop at Stage 1 if the required cumulative response of both stages has already been achieved. It has been shown 23 that a study can stop early for a go decision if it is designed to test a null hypothesis only.

Let $X_{1}^{-}$ and $X_{1}^{+}$ be the number of responders in Stage 1 for the biomarker‐negative and biomarker‐positive sub‐populations, respectively. When there is no enrichment, let $X_{2}^{-}$ and $X_{2}^{+}$ be the number of responders in Stage 2 for the biomarker‐negative and biomarker‐positive sub‐populations, respectively. When there is enrichment, let $X_{2e}^{+}$ be the number of responders in the biomarker‐positive sub‐population in Stage 2. The total numbers of responders are defined by adding the corresponding responders in each stage, that is, $X^{+}=X_{1}^{+}+X_{2}^{+},X^{-}=X_{1}^{-}+X_{2}^{-}$ and $X_{e}^{+}=X_{1}^{+}+X_{2e}^{+}$.

At Stage 1, $k_{1}^{-}$ and $k_{1}^{+}$ are the minimum number of responders required for each sub‐population, to continue the study. When there is no enrichment, *k* ^−^ and *k* ^+^ are the minimum number of responders for each sub‐population, to declare positive results. When there is enrichment, $k_{e}^{+}$ is then the minimum number of responders for the biomarker‐positive sub‐population at Stages 1 and 2. All *X*'s are binomially distributed, and we have that $k_{1}^{-}\leq k^{-}$ and $k_{1}^{+}\leq k_{e}^{+}$.

At the end of the study, there are three possible positive trial outcomes: rejecting both null hypotheses and claiming efficacy in the unselected population; rejecting $H_{0}^{+}$ and claiming efficacy in the biomarker‐positive sub‐population without enrichment; and rejecting $H_{0}^{+}$ and claiming efficacy in the biomarker‐positive sub‐population after enrichment. Each of these three outcomes are labelled Routes 1, 2 and 3, respectively, in Figure 1.

Note that it is neither a biomarker stratified design (because of the second stage depending upon rules for the first stage based on activity in biomarker‐positive subjects coupled with activity in the biomarker‐negative subjects) nor an enrichment design (because the focus is not only biomarker‐positive subjects) in the true sense. Instead, it is an *adaptive enrichment* design that enriches the biomarker‐positive participants only adaptively conditional on observing a lack of activity in the biomarker‐negative subjects and some activity in the biomarker‐positive subjects. The design can be indexed completely by the 10 design parameters

(1) $$\left(k_{1}^{-}\;\;k_{1}^{+}\right)/\left(N_{1}^{-}\;\;N_{1}^{+}\right)\;\to \;\left(k_{e}^{+}/N_{e}^{+}\right)\;|\;\left(k^{-}\;\;k^{+}\right)/\left(N^{-}\;\;N^{+}\right)$$

where parameters to the left of the arrow are the Stage 1 thresholds (*k*) out of the sample sizes (*N*), while parameters to the right of the arrow are the Stage 2 thresholds out of the respectivesample sizes. Therefore, given the aforementioned 10 design parameters together with the response rate probabilities, the study is completely pre‐specified and ready to be implemented. This leads to simple rules for making decisions at the interim analysis.

## 3 Calculating the Hypothesis Rejection Probabilities

We now look at the probabilities of rejecting the hypotheses and hence determine the significance and power for the study design. It is important to note that the formulae given in the JH paper 20, Equations (5)–(8) do not take into account the dependence between Stage 1 results and the Stage 2 tests. The probabilities for Stage 2 are conditional upon the number of responders at Stage 1, and so, their product should be summed over *i* up to the minimum of $\left(N_{1}^{-},k^{-}-1\right),\left(N_{1}^{+},k_{e}^{+}-1\right)$ instead of just $N_{1}^{-},N_{1}^{+}$ and so on because the maximum number of responders in Stage 1 will either be the total number of responders at the end of Stage 2 or the numbers recruited at Stage 1, whichever is the minimum. The formulae given here express the conditional probabilities of rejecting the hypotheses in both the sub‐populations.

The probability of rejecting both hypotheses $H_{0}^{-}$ and $H_{0}^{+}$ via Route 1 (Figure 1), that is, declaring a go decision in the *unselected* population, is

(2) $$R_{1}(p^{-})\;=\;\left(\overset{\min\left(N_{1}^{-},k^{-}-1\right)}{\underset{i=k_{1}^{-}}{\sum }}\;\;P\left(X_{2}^{-}\;\geq \;k^{-}\;-i\right)P\left(X_{1}^{-}\;\;=\;i\right)\;\right)\;+\;P\left(X_{1}^{-}\;\geq \;k^{-}\right).$$

Note that this formula is different from Equation (5) of 20. The first term of (2) represents the probability that the responders at Stage 2 are greater than or equal to the required responders at Stage 2 conditional on the cut‐off responders at Stage 1, with appropriate summation as mentioned earlier. The additional second term in (2) yields the probability that the number of responders at Stage 1 itself is greater than the cumulative responders required at the end of the second stage. Note that *R*_1_(*p* ^−^) is a monotonically increasing function of *p* ^−^, the response rate in the negative population, and also that rejecting both null hypotheses does not depend on the response in the positive sub‐population.

The probability of rejecting $H_{0}^{+}$ via route 2 (Figure 1), that is, declaring a go decision in the *biomarker‐positive* sub‐population, is

(3) $$R_{2}(p^{-},p^{+})\;=\;P(X^{+}\;\geq \;k^{+})\left(\overset{\min(N_{1}^{-},k^{-}-1)}{\underset{i=k_{1}^{-}}{\sum }}\;\;P(X_{2}^{-}\;\;<\;k^{-}\;\;-i)P(X_{1}^{-}\;\;=\;i)\right)$$

Note that *R*_2_(*p* ^−^,*p* ^+^) is a monotonic function of *p* ^+^ but for fixed *p* ^+^ the function is not monotonic in *p* ^−^ and has a single maximum, a formula for which is given in the Supporting Information.

The probability of rejecting $H_{0}^{+}$ via Route 3 (Figure 1), that is, declaring a go decision in the *biomarker‐positive* sub‐population with enrichment, is

(4) $$\begin{array}{c} R_{3}(p^{-},p^{+})=P(X_{1}^{-}<k_{1}^{-})\left(\left\{\overset{\min(N_{1}^{+},k_{e}^{+}-1)}{\underset{i=k_{1}^{+}}{\sum }}P(X_{2e}^{+}\geq k_{e}^{+}-i)P(X_{1}^{+}=i)\right\}+P(X_{1}^{+}\geq k_{e}^{+})\right) \end{array}$$

Formulae (3) and (4) are also different from Equation (6) of 20, and take into account the conditional probabilities. Equation (4) has an additional term which represents the probability that, for the *biomarker‐positive* sub‐population, the number of responders at Stage 1 itself is greater than the required cumulative responders at both stages. Note that *R*_3_(*p* ^−^,*p* ^+^) is a decreasing function of *p* ^−^ and an increasing function of *p* ^+^. The probability of obtaining a positive result in Equation (2) only depends on the true response rate *p* ^−^ in the negative population, while the other routes of obtaining a positive result (via Equations (3) and (4)) depend on the true response rates (*p* ^−^,*p* ^+^) in both the subgroups.

In order to evaluate these probabilities, we will assume the responders, *X*'s, follow binomial distributions: $X^{+}∼B(N^{+},p^{+}),X_{2}^{-}∼B\left(N_{2}^{-},p^{-}\right),X_{1}^{-}∼B\left(N_{1}^{-},p^{-}\right),X_{2e}^{+}∼B\left(N_{2e}^{+},p^{+}\right)$ and $X_{1}^{+}∼B\left(N_{1}^{+},p^{+}\right)$.

From these functions, we can denote the total probability of rejecting $H_{0}^{+}$ via Routes 2 or 3 as *R*_23_(*p* ^−^,*p* ^+^) = *R*_2_(*p* ^−^,*p* ^+^) + *R*_3_(*p* ^−^,*p* ^+^). Also, we can denote the total probability of rejecting at least one null hypothesis as *R*_123_(*p* ^−^,*p* ^+^) = *R*_1_(*p* ^−^) + *R*_2_(*p* ^−^,*p* ^+^) + *R*_3_(*p* ^−^,*p* ^+^). Using the pre‐specified targeted response rates for each sub‐population, $p_{1}^{-}$ and $p_{1}^{+}$, we consider three different scenarios: no efficacy, $\left(p_{0}^{-},p_{0}^{+}\right)$; efficacy in the unselected population, $\left(p_{1}^{-},p_{1}^{-}\right)$; and efficacy in the biomarker‐positive sub‐population only, $\left(p_{0}^{-},p_{1}^{+}\right)$. Table 1 summarises the probability of positive trial outcomes for each scenario, where *R*_0_(*p* ^−^,*p* ^+^) = 1−*R*_123_(*p* ^−^,*p* ^+^) is the probability of no positive outcome, that is, not rejecting any of the null hypotheses.

**Table 1:** The probability of each positive outcome at three pre‐specified real‐world scenarios.

	Outcomes		
Real world	No Efficacy	Reject $H_{0}^{-}$ and $H_{0}^{+}$	Reject $H_{0}^{+}$
1. No Efficacy $\left(p_{0}^{-},p_{0}^{+}\right)$	$R_{0}\left(p_{0}^{-},p_{0}^{+}\right)$ True negative	$R_{1}(p_{0}^{-})$ False positive	$R_{23}\left(p_{0}^{-},p_{0}^{+}\right)$ False positive
2. Unselected $\left(p_{1}^{-},p_{1}^{-}\right)$	$R_{0}\left(p_{1}^{-},p_{1}^{-}\right)$ False negative	$R_{1}\left(p_{1}^{-}\right)$ True positive	$R_{23}\left(p_{1}^{-},p_{1}^{-}\right)$ Wrong positive
3. Positive only $\left(p_{0}^{-},p_{1}^{+}\right)$	$R_{0}\left(p_{0}^{-},p_{1}^{+}\right)$ False negative	$R_{1}\left(p_{0}^{-}\right)$ Wrong positive	$R_{23}\left(p_{0}^{-},p_{1}^{+}\right)$ True positive

The notion of *Wrong Positives* in the aforementioned table is where one rejects the null hypothesis for the biomarker‐positive sub‐population when the effect is in the unselected and where one rejects both hypotheses when the effect is in the positive sub‐population only.

### 3.1 Power

The probability of rejecting both null hypotheses for the *unselected* scenario is $R_{1}(p_{1}^{-})$. In other words, this is the power for the *unselected* subgroup via Route 1 assuming the true response was $\left(p_{1}^{-},p_{1}^{-}\right)$. The probability of rejecting $H_{0}^{+}$ only for the biomarker‐ positive only scenario is $R_{23}\left(p_{0}^{-},p_{1}^{+}\right)$. In other words, this is the power of concluding a positive outcome in a *biomarker‐positive* patient population assuming the true responses are $\left(p_{0}^{-},p_{1}^{+}\right)$.

The desired power for this trial design is either a high probability of rejecting both hypotheses for the unselected scenario or there is a high probability of rejecting the biomarker‐positive null hypothesis for the biomarker‐positive only scenario. Allowing for the smaller of the two powers, we recommend that the overall power is

(5) $$\min\left(R_{1}\left(p_{1}^{-}\right),R_{23}\left(p_{0}^{-},p_{1}^{+}\right)\right)$$

### 3.2 Type I error control

Because we have more than one null hypothesis, the familywise error rate (FWER) needs to be controlled. Our family of null hypotheses is $\left\{H_{0}^{+},H_{0}^{-}\right\}$, and let *V*={0,1,2} be the number of true null hypotheses that are rejected at the end of the adaptive trial. We require that FWER = P(V≥1)≤α. Table 2 shows, for the three allowable parameter constellations and rejection decisions, the type I error and power constraints of the proposed design. The value of *V* is shown for each case in bold brackets.

**Table 2:** Power, Type I error constraints and the value of *V* for each design scenario and rejection decision.

Scenario	Reject $H_{0}^{-}$ and $H_{0}^{+}$	Reject $H_{0}^{+}$	Constraint
	*R*_1_(*p* ^−^)	*R*_23_(*p* ^−^,*p* ^+^)	
1. $\left(p_{0}^{-},p_{0}^{+}\right)$	≤*α* **(2)**	≤*α* **(1)**	∑≤α
2. $\left(p_{1}^{-},p_{1}^{-}\right)$	≥ power **(0)**	[]**(0)**	—
3. $\left(p_{0}^{-},p_{1}^{+}\right)$	≤*α* **(1)**	≥ power **(0)**	—

Power is defined as in Equation (5), and the Type I error is given by

(6) $$R_{123}\left(p_{0}^{-},p_{0}^{+}\right)\leq \alpha .$$

Equation (6) may make it appear that we are only controlling FWER in the weak sense, which is when all null hypotheses are true. However, by doing so, we are also controlling the probability of incorrectly rejecting $H_{0}^{-}$ and correctly rejecting $H_{0}^{+}$ when $p^{-}\;=\;p_{0}^{-}$ and $p^{+}\;\;=\;p_{0}^{+}$. This is because *R*_1_(*p* ^−^) is independent of the value of *p* ^+^. Hence, control is also in a strong sense. Note that nothing is specified about controlling the rate of wrong positives and we ignore individual weighting of each positive outcome.

### 3.3 Expected sample size

Let us now define the *expected sample size* and the associated optimality criteria for this design. If the trial stops at the first stage, the sample size is *N*_1_. If the trial continues to the second stage, then the sample size will either be *N*_1_+*N*_2_ or $N_{1}+N_{2e}^{+}$. The expected sample size is therefore

(7) $$E(N)\;=\;\;N_{1}+N_{2}P\left(k_{1}^{-}\;\;\leq \;X_{1}^{-}\;<\;k^{-}\right)+N_{2e}^{+}P\left(X_{1}^{-}\;\;<\;\;k_{1}^{-}\right)P\left(k_{1}^{+}\;\;\leq \;X_{1}^{+}\;\;<\;k_{e}^{+}\;\right).$$

Let Ω be the set of all designs that satisfy the Type I error constraint and have sufficient power. Then, the optimal design is an element of Ω that has the smallest expected sample size *E*(*N*) under the global null hypothesis $\left(p^{-},p^{+}\right)=\left(p_{0}^{-},p_{0}^{+}\right)$, where $X_{1}∼B\left(N_{1}^{-},k_{1}^{-}-1,p^{-}\right)$ and $X_{1}^{+}∼B\left(N_{1}^{+},k_{1}^{+}-1,p^{+}\right)$. Formula (7) now takes into account early stopping for efficacy. The overall probability of early termination *PET* is given by the formula

(8) $$\mathrm{PET}\;=\;P\left(X_{1}^{-}\;\geq \;k^{-}\right)+P\left(X_{1}^{-}\;\;<\;k_{1}^{-}\right)\left[P\left(X_{1}^{+}\;\geq \;k_{e}^{+}\right)\;+\;P\left(X_{1}^{+}\;\;<\;k_{1}^{+}\right)\right]$$

## 4 Results

We now present the results for the operating characteristics due to JH, as well as our new optimal designs.

### 4.1 Jones–Holmgren tables revisited

In Table 3, we show the route probabilities (corresponding to power and Type I error rates calculated using Formulae (2)–(4)) and expected sample sizes for the same parameter constellations as in Table 1 of Jones and Holmgren 20. Note that the power in the biomarker‐positive sub‐population differs when calculated using our formulae. We also explicitly give the expected sample sizes, both due to the two parallel Simon two‐stage design *E*(*N*)_*Simon*_ (defined in Appendix A of 20) as well as the adaptive design *E*(*N*)_*Adaptive*_.

**Table 3:** Operating characteristics given the design (2 1)/(34 14) → (5/50) | (4 4)/(53 27).

						*E*(*N*)_*Adaptive*_ /
$p_{1}^{-}$	$p_{1}^{+}$	$R_{1}(p_{1}^{-})$	$R_{23}(p_{0}^{-},p_{1}^{+})$	*E*(*N*)_*Simon*_	*E*(*N*)_*Adaptive*_	*E*(*N*)_*Simon*_
0.03	0.03	0.067	0.012	74.61	65.79	0.881
0.03	0.10	0.067	0.424	85.21	76.91	0.902
0.03	0.15	0.067	0.720	88.36	80.21	0.907
0.10	0.15	0.755	0.720	127.66	80.03	0.626
0.10	0.25	0.755	0.905	129.78	80.44	0.619
0.15	0.30	0.952	0.924	136.99	80.10	0.584

$p_{0}^{-}=p_{0}^{+}=0.03$, Significance, *α* = 0.079

For power, let us consider the targeted response rates $p_{1}^{+}=0.15,p_{1}^{-}=0.10$ from Table 3. The probability of rejecting both hypotheses (i.e. Route 1) is 75.5*%*, which is the same as that obtained by JH. Now, the probability of rejecting $H_{0}^{+}$ (i.e. Routes 2 and 3) is quoted in JH as 17.5*%* making the overall power of 93*%* as per their definition (Equations (7) and (8) of 20) of adding these rejection probabilities at different response rates. However, using the formulae as described in the preceding section yields a probability of 72*%*, and we claim the power of their design is therefore 72*%* (the minimum of the two rejection probabilities), that is, less than the desirable 80*%*.

In the next subsection, we exhibit the optimal designs obtained using the formulae given in the previous section.

### 4.2 Optimal designs

In the previous section, we have already explained what we mean by optimal designs. A point to note is that Simon's optimal design is under the assumption that the null hypothesis is true. Of late, there has also been interest in generating optimal design strategies under the alternative hypothesis 23, 24, 25; however, we shall not delve into this aspect in the current paper. Note that the expected sample sizes obtained in Table 3 are not optimal. This is in contrast to our method for obtaining the designs where we choose the one with the smallest expected sample size and present the associated design parameters. Table 4 below gives optimal designs for various different sets of the targeted response probabilities and controlling the FWER. The null hypotheses set $p_{0}^{-}=p_{0}^{+}=0.03$.

**Table 4:** Optimal designs — controlling FWER at 5*%* and setting $p_{0}^{-}=p_{0}^{+}=0.03$.

			$R_{1}(p_{1}^{-})$	$R_{23}(p_{0}^{-},p_{1}^{+})$			Optimal design
$p_{1}^{-}$	$p_{1}^{+}$	Significance	(unselected)	(positives)	PET	*E*(*N*)	$\left(k_{1}^{-}k_{1}^{+})/(N_{1}^{-}N_{1}^{+})\to (k_{e}^{+}/N_{e}^{+})\;|\;(k^{-}k^{+})/(N^{-}N^{+}\right)$
0.10	0.10	0.048	0.800	0.800	0.623	110.2	(3 2)/(44 34) → (7/104) | (9 4)/(135 53)
0.10	0.15	0.049	0.801	0.801	0.653	77.9	(2 2)/(32 21) → (6/67) | (7 3)/(106 29)
0.10	0.25	0.050	0.800	0.800	0.571	60	(2 1)/(34 8) → (4/29) | (6 2)/(87 9)
0.15	0.15	0.050	0.802	0.801	0.611	46.9	(2 1)/(20 12) → (4/43) | (6 2)/(66 21)
0.15	0.25	0.046	0.803	0.802	0.561	32.5	(1 1)/(12 7) → (4/28) | (4 2)/(43 11)
0.15	0.35	0.045	0.801	0.800	0.615	27.8	(1 1)/(11 5) → (3/15) | (4 2)/(47 7)
0.25	0.25	0.045	0.802	0.801	0.695	18.5	(1 1)/(6 6) → (3/24) | (3 2)/(23 13)
0.25	0.40	0.038	0.802	0.801	0.742	13.5	(1 1)/(6 4) → (2/9) | (3 2)/(23 5)

The optimal designs were calculated by an exhaustive search over the 10‐dimensional design parameter space. This space is very large, containing up to 10^17^ possible designs for the larger trials needed for low‐targeted responses. To make the computation tractable, the search space was pruned wherever possible, using strictly logical (i.e. non‐heuiristic). For example, the power in the unselected population can be calculated using only four parameters, and if the power is too small, we do not need to iterate over the remaining six. This can reduce the search space by perhaps three orders of magnitude, depending on the parameters.

The program was run using a Graphics Processing Unit, or GPU, similar to the graphics card in many high‐end computers. A GPU contains several hundred small processors and is suitable for massively parallelisable problems, like this one, where each possible design can be calculated in parallel. The GPU provides a gain in speed of between 5 times and 50 times, depending on the parameters used. These techniques reduced the program execution time to between 30 s and 24 h, with the longer times required when the expected sample size (and hence the search space) was large. The maximum size of the search space needs to be configured by the user, but it is easy to set a search space sufficiently larger than the proposed optimal design to be confident that it is indeed the true optimum. The code is available at the weblink http://www.mrc-bsu.cam.ac.uk/published-research/additional-material/.

The designs in Table 4 have been obtained by fixing the significance level to be at most 5*%* and power to be at least 80*%*. Comparing the example trial *p* ^+^=0.15,*p* ^−^=0.10 used by JH from Table 3 with *E*(*N*)_*Simon*_=127, we find that our optimal designs for these response rates offer a substantial efficiency in terms of the expected sample sizes of 78. According to our definition, this design also has a smaller expected sample size than the design suggested by Jones and Holmgren that had insufficient power. Our designs yield even smaller expected sample sizes as we increase the desired response rates to be much higher than the null response rates of 3*%*. All across Table 4, the probability of declaring a go decision for the unselected population at Stage 1 remains very low with $P\left(X_{1}^{-}\geq k^{-}\right)=5.04\times 10^{-4}$ being the maximum.

**Figure 2:**
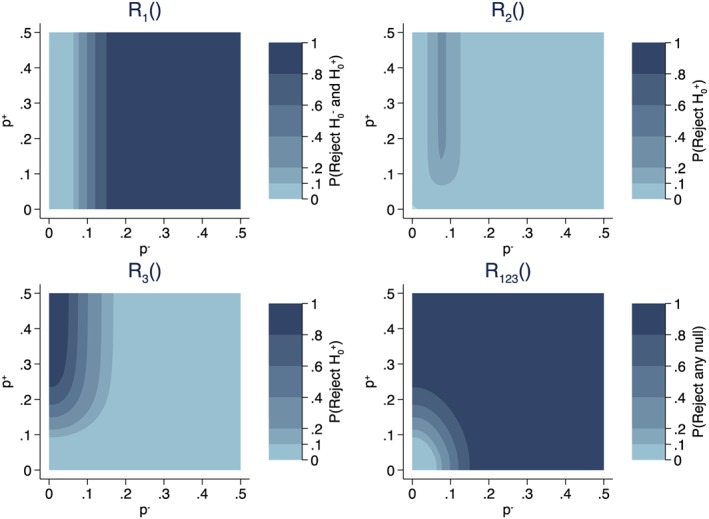
The rejection probabilities for each route.

The rejection probability functions are plotted in Figure 2 for the design from the first row of Table 4, (3 2)/(44 34) →(7/104) |(9 4)/(135 53).

As shown earlier, the function *R*_2_() is non‐monotonic in *p* ^−^ but monotonic in *p* ^+^. Using our design‐finding software, one can obtain a plethora of optimal designs by varying the null and the desired response rate probabilities. A selection of these is available at the aforementioned URL.

## 5 Discussion

In this article, we have taken the design of Jones and Holmgren and provided alternative definition of power and choice of Type I error controls. Additionally, we introduce an extension of their work to provide designs that are optimal, in the sense that we are minimising the expected sample size. A selection of optimal designs are provided in the Supporting Information including the computer code to create any design required. We demonstrated that our optimal design was more efficient than Jones and Holmgren's original, and also it gave a 60*%* reduction in the expected sample size compared to the parallel Simon two‐stage design. Table 5 summarises our work against that of Jones and Holmgren.

**Table 5:** Comparison summary of JH and our work

JH design	Our version
Adaptive enrichment with futility stopping	Adaptive enrichment with futility and go‐decision stopping
Rejection probabilities not conditional on Stage 1 results	Rejection probabilities conditional on Stage 1 results
Formula for total probability of rejecting at least one null,	*R*_123_ has terms evaluated at same response rates
*R*_123_, has terms evaluated at different response rates	
*Overall power:* *R*_123_	*Overall power:* min(*R*_1_,*R*_23_)
Characterises the operating characteristics of the	Aims to control the type I and type II error rates, and
procedure without explicit control of Types I and II	several options for weak and strong FWER exist
error rates	
*Method used to obtain designs:* Fix design parameters and	*Method used to obtain designs:* Fix Type I error and power
investigate the operating characteristics of procedure until	constraint; algorithmic search yields optimal designs
a satisfactory design is found	

Underlying assumptions common to both: $p_{0}^{-}\;=\;p_{0}^{+}$(no prognostic effect), $p_{1}^{-}\;\leq \;p_{1}^{+}$(order restriction)

JH, Jones and Holmgren.

The underlying assumption $p_{0}^{-}=p_{0}^{+}$ may signify that there is no prognostic effect. Because a trial design cannot distinguish a prognostic biomarker from a predictive one, we assume that the biomarker is predictive. However, the biomarker could be prognostic too, but we have not attempted to evaluate this. The optimal designs obtained in our paper are, however, robust to deviations from this assumption because in our programme code one can *a priori* specify the different values of $p_{0}^{-}$ and $p_{0}^{+}$.

Another major assumption of the Jones and Holmgren design is the order restriction on the parameter space, that is, the response for the biomarker‐positive sub‐population is bigger than in the biomarker‐negative sub‐population. One implication of this is when the $H_{0}^{-}$ hypothesis is rejected then both are rejected without using the information in the biomarker‐positive sub‐population, which represents an inefficient use of the data. Another, more fundamental issue is that even if expert scientific opinion suggest biomarker status rigidly dictates treatment response, the assumption could be wrong. Note that if the order restriction is relaxed then an additional wrong positive error may occur. This is the case of only rejecting $H_{0}^{+}$ for the additional scenario of an effect in the negative subgroup only $\left(p_{1}^{-},p_{0}^{+}\right)$.

It is widely known that single‐arm trials may be subject to selection bias and any treatment response being due to the patient population rather than the effect of treatment. Additionally, for the stratified single‐arm trials, a positive result might mean that the biomarker is a prognostic biomarker rather than a predictive biomarker. A randomised trial will be needed to confirm predictive ability; however, a single‐arm trial is much smaller and could be valuable within a drug development plan. Recent literature 26, 27, 28 on single‐arm trials in oncology continues to provide early indication of effectiveness of the interventional drug, for example, in the evaluation of cytotoxic treatment resulting in tumour reduction. Given that the goal of single‐arm trials is hypothesis testing, they screen out ineffective drugs quickly and cheaply. Such trials are also of benefit where the goal is to prioritise which, if any, experimental regimen should progress to Phase III when there is no *a priori* information to favour one. Useful contexts include Phase II selection designs (of two or more parallel single‐arm studies) when selecting among new agents, among different schedules or doses. An extension of our work would lead to a randomised adaptive enrichment with endpoints being response, progression‐free survival or overall survival. It is worth comparing this with other adaptive enrichment design approaches. In 29, Wang *et al*. adaptation is about sample size and futility stopping, and the testing leads to a mixture of treatment effects thus making the trial results challenging to interpret. Jenkins *et al*. 30 use endpoints at the interim and the end of the trial that are different but correlated. Their adaptation pertains to selection of treatment arms, while in our enrichment design, the trial continues seamlessly either in the biomarker‐positive sub‐population or in the general unselected population based on the data obtained at Stage 1 with a single overall primary endpoint.

For the original Simon two‐stage design, the function of rejecting the null hypothesis, say *R*(), was monotonic. This allows the null hypothesis to be specified as an interval *H*_0_:*p*≤*p*_0_ rather than a single‐point null hypothesis. Also, it meant that there is sufficient power for any response greater than the target response. However, in our stratified version, *R*_2_(*p* ^−^,*p* ^+^) is a non‐monotonic function, and hence, *R*_123_(*p* ^−^,*p* ^+^) is non‐monotonic. Therefore, the specification of a range null hypothesis $H_{0}^{-}:p\leq p_{0}^{-}$ is difficult because either of the error rates could increase or decrease, and so, the theory may not be robust, and the designs thus obtained may not be reliable. However, it is possible to see from the plots in Figure 2 that there is a region of ‘null’ responses that have sufficient FWER control and sufficient power is obtained for a wide range of targeted responses. At this point, it is worth comparing our hypotheses setup with that of Zhong 31 where he formulates the null hypothesis *H*_0_:*p* = *p*_*c*_ with *p*_*c*_ being the minimal effective response rate, and the alternative hypotheses are $H_{1}^{n}:p<p_{c},H_{1}^{g}:p>p_{c}$ signifying a no‐go decision and a go decision, respectively. However, we cannot have such inequalities in our alternative hypotheses for the stratified design because of the reasons of non‐monotonicity mentioned previously.

When obtaining our optimal designs, we did not attempt to control the rate of wrong positives, and we ignored the individual weighting of each positive outcome. Of course, one may wish to do so. In the Supporting Information, we discuss several alternative methods of error control and present alternative tables of optimal designs that flow from them. This is available at the aforementioned URL.

It might not be possible to plan the enrolment of precise numbers of biomarker‐positive and of biomarker‐negative participants. In future work, we plan to expand our algorithm to compute optimal designs providing overall sample sizes only without the need to find fixed number of biomarker‐positive or negative samples. Effectively, the algorithm needs to integrate out all possibilities of biomarker‐positive and negative sample sizes given an overall size. Because our algorithm is combinatorial in nature, essentially enumerating all possible scenarios, such integration can be easily incorporated. That is, given a fixed prevalence rate in addition to the other parameters described earlier, the algorithm will provide a design not in terms of biomarker‐positive and negative sample sizes but overall sample sizes. Such extension is easily incorporated by keeping track of modified expected sample size calculations based on the binomial distribution using the biomarker prevalence rate. A branch and bound approach for small probability regions of the combinatorial space will allow us to cut down the search space.

A subtle point is that absolute fulfilment of false positive and false negative constraints can no longer be guaranteed. If only total sample sizes are given, even with non‐extreme biomarker prevalence rates, low numbers of biomarker‐positive or negative sample sizes with high error rates are possible, if unlikely. However, exploiting the low probability of such cases, guarantees can be provided for the proposed designs to breach error rate constraints with only a small and user defined probability.

## Supporting information

Supporting info item
