# Supplementary material for: An optimal stratified Simon two‐stage design
Source: Pharm Stat. 2016 Mar 2;15(4):333–40. doi: 10.1002/pst.1742 (PMC5405342; doi:10.1002/pst.1742)
Supplement: Supplementary file 1 — Supporting info item [file PST-15-333-s001.pdf]

## Monotonicity of $R_2(p^-, p^+)$

The probability of declaring the treatment effective via Route (2), $R_2(p^-, p^+)$, is:

$$P(X^+ \geq k^+) \left( \sum_{i=k_1^-}^{min(N_1^-, k^- - 1)} P(X_2^- \leq k^- - i - 1) P(X_1^- = i) \right)$$

$R_2(p^-, p^+)$ is a monotonic increasing function of $p^+$ but not a monotonic increasing or decreasing function of $p^-$. Indeed, it will be maximised for a particular value of $p^-$. To see this we plot the inner summation term, representing the probability of event $(X_1^- \geq k_1^-, X_1^- + X_2^- \leq k^- - 1)$, for a real data example. Let $(N_1^-, N_2^-, k_1^-, k^-)$ = (20,20,3,7). Figure 3 shows this probability as a function of $p^-$.

The dotted vertical line shows the value of $p^-$ that maximises this probability. We can in general calculate the maxima by differentiating the expression with respect to $p^-$ and finding the value that sets it to zero. We first rewrite this as

$$R_2^*(p^-) = \sum_{i=k_1^-}^{min(N_1^-, k^- - 1)} B(N_2^-, k^- - i - 1, p^-) b(N_1^-, i, p^-)$$

where $b(.)$ and $B(.)$ represent the binomial mass function and the cumulative distribution function, respectively, of the relevant binomial distribution. Using the fact that

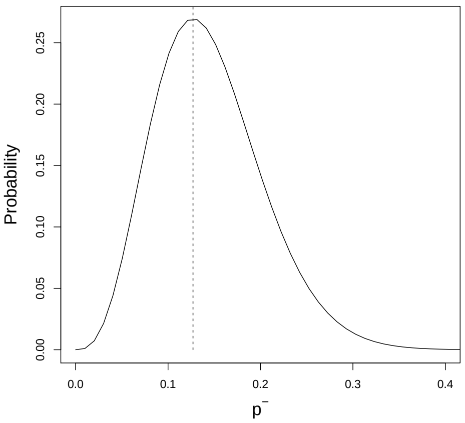


Figure 3: $R_2(p^-, p^+)$ *as a function of* $p^-$ *for a fixed* $p^+$.

$$\begin{aligned}
\frac{dB(N_2^-, k^- - i - 1, p^-)}{dp^-} &= -\frac{(N_2^- - k^- + i + 1)}{1 - p^-} b(N_2^-, k^- - i - 1, p^-) \\
\frac{db(N_1^-, i, p^-)}{dp^-} &= \left(\frac{i - N_1^- p^-}{p^-(1 - p^-)}\right) b(N_1^-, i, p^-)
\end{aligned}$$

we differentiate $R_2^*(p^-)$ by parts to give

$$\begin{aligned}
\frac{dR_2^*(p^-)}{dp^-} = \sum_{i=k_1^-}^{min(N_1^-, k^- - 1)} b(N_1^-, i, p^-) \quad \Bigg( \quad & \left(i - N_1^- p^-\right) B(N_2^-, k^- - i - 1, p^-) \\
- \quad & p^- \left(N_2^- - k^- + i + 1\right) b(N_2^-, k^- - i - 1, p^-)\Bigg)
\end{aligned}$$

One can see from Figure 3 that $\frac{dR_2^*(p^-)}{dp^-} = 0$ for ($p^- \approx 0.127$).
